# Application of nanoparticles in antibody drug delivery

**DOI:** 10.3389/fbioe.2026.1759915

**Published:** 2026-02-05

**Authors:** Wumiti Xiefukaiti, Qingmei Zhu, Nadira Dolkun, XueJun Xiao

**Affiliations:** 1 Department of Pharmacology, Xinjiang Medical University, Urumqi, China; 2 Institute of Materia Medica, Xinjiang Medical University, Urumqi, China

**Keywords:** biomaterials, drug delivery systems, monoclonal antibody, nanobody, nanoparticles

## Abstract

Due to their special molecular weight and pharmacokinetic/pharmacodynamic (PK/PD) characteristics, antibody drugs have difficulty crossing the body barrier and entering complex microenvironments to provide effective treatment for encephalopathy, cancer, and severe infectious diseases. With the development and application of nanotechnology, drugs can be modified by nanomethods to increase the delivery efficiency, targeting, and permeability, reduce the resistance to the antibody, and improve the response rate, thus increasing their safety and effectiveness. Therefore, nanoparticles have extensive research and application value in drug delivery systems. This article summarizes the characteristics of nanoparticles and nanoantibody delivery systems to provide a reference for the future application of nanoparticles in drug delivery.

## Introduction

Monoclonal antibodies and other antibody-based therapeutics have emerged as cornerstone treatment modalities in contemporary biomedicine, owing to their highly specific targeting capabilities and therapeutic potential. However, the clinical delivery of these macromolecular drugs remains significantly challenging. Their large molecular weight and hydrophilic nature hinder efficient traversal across critical physiological barriers, such as the blood–brain barrier, the placental barrier, and dense tumor stroma. Additionally, antibody therapeutics are characterized by limited tissue penetration, rapid systemic clearance, potential immunogenicity, and a predominant reliance on parenteral administration routes. These factors collectively constrain their effective concentration and therapeutic efficacy within target tissues. Consequently, the development of novel delivery systems capable of overcoming these obstacles is paramount for enhancing the clinical performance of antibody drugs.

Within this context, nanotechnology offers a promising strategy for optimizing antibody drug delivery. Nanotechnology is dedicated to the study of material properties and applications at the scale of 1–100 nm, serving as a pivotal interdisciplinary field bridging materials science, physical chemistry, and biomedicine (Madkour, 2019). Drug carriers engineered through nanotechnology leverage their unique nanoscale effects, functionalizable surface properties, and tunable physicochemical characteristics to effectively load, protect, and directionally transport antibody molecules. This enables achieving multiple objectives, including precise targeting, controlled release, and enhanced barrier penetration. With the continuous advancement of research, traditional delivery systems are increasingly inadequate in meeting the clinical demand for the precise delivery of macromolecular drugs, particularly antibody-based agents. This further underscores the critical importance and urgency of developing nano-engineered delivery platforms.

## Characteristics and applications of nanoparticles

### The characteristics of nanoparticles

**FIGURE 1:**
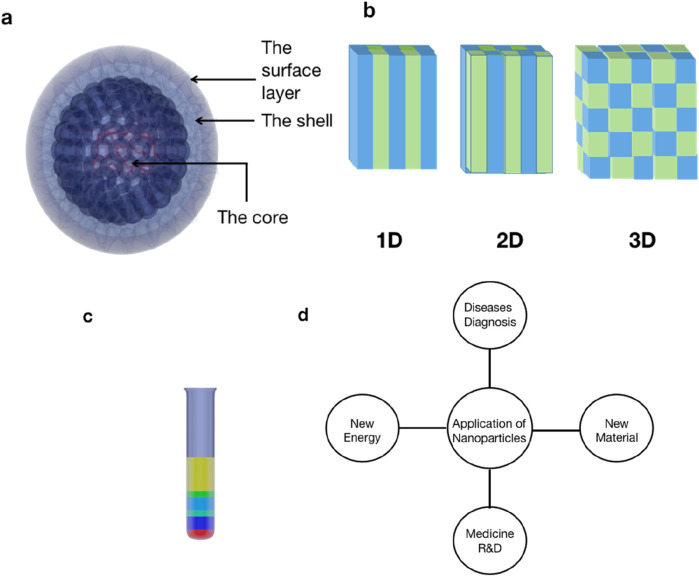
Structure of a nanoparticle. **(a)** The nanoparticle consists of three layers: the surface layer, which can be functionalized with various small molecules, metal ions, surfactants, and polymers; the shell, which is a chemical material different from the core in all aspects; and the core, which is essentially the central part of the nanoparticle. **(b)** Nanoparticles have different structures because of their different size and combinations. **(c)** The aspect ratio and the thickness of the nanoshell determine the size of nanoparticles, and the specific surface area and metal concentration determine their absorption in the visible region. The change in any of the above factors will affect the absorption characteristics of nanoparticles, so different absorption wavelengths are observed. **(d)** Nanoparticle applications mainly focus on the biomedical field for drug delivery systems and disease diagnosis.

Nanoparticles vary in terms of size and structure. They can be considered as 0D, 1D, 2D, or 3D structure (Tiwari et al., 2012). A nanoparticle consists of three layers: the surface, shell, and core. The surface determines the wavelengths that could be detected by imaging (Shin et al., 2016; Khan et al., 2019). The surface layer contains different groups, which divide the nanoparticles into fullerene, metal nanoparticles, ceramic nanoparticles, and polymer nanoparticles. Their reactivity, toughness, and other properties also depend on their unique size, shape, and structure. Because of these characteristics, they are used in catalysis, imaging, and medical treatment (Figures 1a–c).

### Application of nanoparticles in disease diagnosis

In the biomedical field, nanotechnology plays an important role in disease diagnosis, drug design, and manufacturing (Paluszkiewicz et al., 2021) (Figure 1d). Nanoparticles can be used as developers and capsule drugs for disease diagnosis. Researchers designed a new phototherapeutic agent that has optical characteristics in the biological window of the electromagnetic spectrum. The newly synthesized phthalocyanine dye is encapsulated in biocompatible protein nanoparticles, so as to realize the dual effects of targeted fluorescence imaging and synergistic therapy of ovarian cancer. The nanopreparation has good biocompatibility, enhances the biological stability and photothermal activity of water, and has high active oxygen generation efficiency (Borlan et al., 2021). After the nanoparticles are discharged into lymph nodes, they are phagocytized and dissociated in the acidic phagosome of inflammatory macrophages, resulting in near-infrared luminescence. pH-amplified self-luminescent near-infrared nanoparticles, which integrate chemiluminescence resonance energy transfer (CRET) and signal amplification strategy, can accurately identify and locate metastatic sentinel lymph nodes (Wang Zenghui et al., 2021). Quantum dots (QDs) were encapsulated in self-assembled C (RGDyk)-poloxamer-188 polymer nanoparticles (NPs) as fluorescent probes to construct glioma-targeted quantum dots-c(RGDyk) NPs, which can be used for image-guided surgical resection of glioblastoma (Wu et al., 2020). The development of melanin nanoparticles in a magnetic resonance imaging (MRI) contrast agent can significantly enhance the MRI signal *in vivo*. It is a kind of contrast agent with great research potential (Chen et al., 2020). Metal shell (AU) dielectric core (BaTiO) nanoparticles smaller than 100 nm can be used for bimodal imaging and photothermal therapy (Wang et al., 2018). Plectin-1 targeted multifunctional nanoparticle probes for pancreatic cancer imaging and can be used with various imaging devices for pancreatic cancer detection (Chen et al., 2018). It can also be used in medical cosmetic materials, such as nano-hydroxyapatite, coating materials for titanium implants, remineralization therapeutic agents for dentin hypersensitivity, or bone regeneration materials (Bordea et al., 2020). Fetal growth restriction is closely related to the impairment of placental function. Nanotechnology to enhance placental function includes targeted injection of the placental growth factor (PlGF) pathway to increase the number of placental terminal villi and the available surface area for nutrition and oxygen intake. Another option is stimulating the target of the mammalian rapamycin pathway to increase nutrient transporters. Animal experiments showed that the weight of fetuses with growth restriction in a mouse model was significantly improved after injecting insulin-like growth factor-2 (IGF-2) that was packaged with iRGD liposome nanoparticles. The conclusion suggests that a dysfunctional placenta can be targeted by nanoparticles to address the growth restriction phenotype (Pritchard et al., 2021).

### Application of nanoparticles in drug delivery systems

**FIGURE 2:**
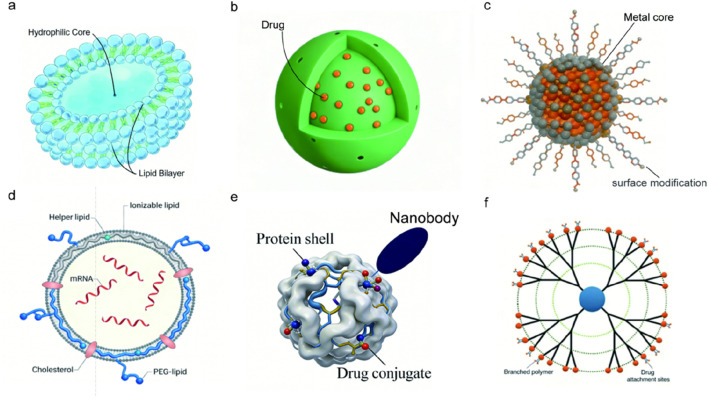
Schematic illustration of common types of nanoparticles used in antibody drug delivery. **(a)** Liposomes—spherical vesicles with a hydrophilic core and lipid bilayer membrane. **(b)** Polymeric nanoparticles—solid matrices formed from biodegradable polymers. **(c)** Metal nanoparticles—inorganic cores with surface functionalization. **(d)** Lipid nanoparticles (LNPs)—ionizable lipid-based systems for nucleic acid delivery. **(e)** Protein/albumin nanoparticles—natural protein-based carriers with high biocompatibility. **(f)** Dendrimers—highly branched, monodisperse polymeric architectures.

The nano-delivery systems currently used in a variety of drug delivery systems include liposomes, polymer micelles, and polymer nanoparticles. As drug carriers, nanoparticles have many excellent properties: protecting drugs from degradation, promoting drug absorption, optimizing pharmacokinetics, improving drug tissue distribution and permeability, and offering good stability under physiological conditions and good tolerance to physiological pressure (Wang et al., 2023). These nanocarrier drug delivery platforms can significantly improve drug delivery efficiency, improve the distribution and target organ concentration of macromolecular drugs *in vivo*, and also improve the targeting efficiency (Figure 2).

#### Drug delivery for central and cardiovascular diseases

Nanoparticle-based drug delivery platforms are increasingly demonstrating their value in the treatment of central and cardiovascular diseases. For example, in central nervous system (CNS) diseases, more and more preclinical studies have proved that the use of brain-targeted nanoparticles can increase the penetration of the blood–brain barrier and improve brain delivery (Mantecón-Oria et al., 2021), which indicates that nanoparticles can be designed as an effective drug delivery system for the treatment of CNS diseases. Studies have confirmed that Tween 80 methoxypoly (ethylene glycol)-poly (lactic acid co-glycolic acid) nanoparticles loaded with rhynchophylline have a higher blood–brain barrier transmission rate and reduce cell death in an *in vitro* model of Alzheimer’s disease. In an *in vitro* model of Parkinson’s disease, trans activated transcripts biologically bound to amphoteric polymer poly (2-methacryloyloxyethyl phosphorylcholine) and protein nanoparticles loaded with non-heme have similar protective ability to free non-heme (Martinez and Peplow, 2022). This shows that nanoparticles can improve the penetration of drugs to the blood–brain barrier and can be used in drug delivery systems for neurodegenerative diseases. VHH-pa2H is the single-domain antibody of Aβ, increasing GSH in the encapsulated liposome and reaching the Aβ section through intravenous injection. The amount of antibody in the plate area increased significantly. At the same time, GSH liposomes can reduce the clearance of antibodies in the blood (Rotman et al., 2015). Building Aβ-targeted nanoparticles can effectively improve the drug concentration in target tissues and increase the blood circulation time of drugs, which can be used to treat Alzheimer’s disease in mice.

In addition to loading nanoparticles with different chemical drugs or plant extracts, nanoparticles can also be designed as drug delivery systems targeting different immune cells to suit the complex needs of targeted drug delivery. For example, monocytes are a population of circulating immune cells with surface pattern recognition receptors (PRRs) that bind to glycoproteins, reactive oxygen species, chemokine receptors, adhesion molecules, and immunoglobulins. Statins are mainly used to treat atherosclerosis. Due to their low bioavailability, they need to be encapsulated with nanoparticles. Statin-loaded HDL nanoparticles can preferentially aggregate in monocyte-derived macrophages rather than in monocyte atherosclerotic plaques (Duivenvoorden et al., 2014). This nano-drug delivery system targeting monocytes loaded with statins can reduce the plaque load of macrophages and reduce the expression of pro-inflammatory (Hossaini Nasr et al., 2020) and monocyte recruitment genes (Gao et al., 2020), which can be used in the treatment of atherosclerosis.

The use of mRNA polymer nanoparticles can effectively improve cardiac function and can be used in the treatment of heart failure (Krienke et al., 2021). The introduction of therapeutic molecules into the damaged myocardium and the delivery of drugs to the “shock” myocardium or the part with dysfunction after transient ischemia of cardiac tissue help to overcome heart failure. Studies have shown that nano-mIR-133a replacement therapy can help alleviate heart failure caused by pressure overload (Modica et al., 2021). PEG-PLGA nanoparticles loaded with mIR-30b-5p can improve cardiac function, reduce myocardial injury, and regulate the expression of factors related to myocardial hypertrophy and inflammation by targeting TGFbR2 (Ren et al., 2021). The superparamagnetic iron oxide (SPIO) nanoparticle-loaded oil-in-water nanoemulsion (NE) for magnetic resonance imaging (MRI) has been shown by dynamic MRI. The NE-SPIO nanoparticles modified with a polyethylene glycol (PEG) layer reduce the uptake in the liver and prolong the half-life. The whole human scFv targeting atherosclerosis can focus on atherosclerotic plaque as a therapeutic tool for atherosclerotic imaging (SPIO) and reduce oxidation in atherosclerotic plaque (Bonnet et al., 2021).

#### Antitumor drug delivery

Because of the high permeability and retention effect (EPR effect) of nanoparticles, they can be better enriched in tumors and inflammatory tissues (Guo et al., 2020). Therefore, nano-delivery systems are often used to target a variety of solid tumors and central nervous system tumors. In one study, lipid nanoparticles composed of G0-C14, PLGA, and lipid PEG were developed and modified with CTCE-9908, a targeting peptide specific for CXCR4 (highly expressed in liver cancer cells), to target liver cancer cells. Using this novel lipid nanoparticle delivery vector to specifically deliver p53 mRNA to liver cancer cells, in combination with an anti-PD-1 monoclonal antibody (mAb), can effectively induce the global reprogramming of tumor microenvironment (TME) and achieve a better antitumor effect (Xiao et al., 2022). Paclitaxel PLGA-PEG nanoparticles have higher targeting and stronger cytotoxicity. They can target and kill CD133-positive lung cancer stem cells (Pang et al., 2022), significantly reduce the tumor volume, and can be used in the treatment of lung adenocarcinoma. Transferrin (TF) biological binding solid lipid nanoparticles (SLNs) can significantly increase the uptake of TF CRC SLNs by tumor cells by loading curcumin (CRC), which is used for active targeted therapy of prostate cancer (Akanda et al., 2021). A Raman-labeled hollow gold nanoparticle has the characteristics of surface-enhanced Raman scattering (SERS). It can accumulate effectively in tumor sites under low-dose X-ray pre-irradiation and play a high antitumor effect in breast cancer models (Qiaolin et al., 2021). Melanoma is the most invasive skin cancer. The metastasis rate of malignant melanoma is very high, and the currently available treatment schemes have difficulty achieving a cure. Paclitaxel (PTX) containing albumin nanoparticles (ANPs) coated with macrophage plasma membrane (RANP) can significantly promote the cytotoxicity and apoptosis rate of melanoma cells, indicating that the albumin nano-drug delivery system coated with macrophage plasma membrane has a significant antitumor effect. It has the potential for the treatment of melanoma (Cao et al., 2020). Studies have confirmed that systematic injection of adriamycin nanoparticles can effectively inhibit tumor growth. Compared with free drugs, tumor cells are more sensitive to small-sized DOX-loaded nanoparticles (Gao et al., 2021). It has been shown that the lipid barrier of a brain tumor is related to the poor prognosis of malignant brain tumors, but the barrier can be penetrated through blood glioma gene therapy (Tavares Luiz et al., 2021).

Nanoparticles can regulate cell function, affect the cellular composition of the microenvironment, exert high targeting and permeability, and inhibit the occurrence and development of tumors. The nano-drug delivery system is used to regulate components such as fibroblasts, blood vessels, and extracellular matrix in the microenvironment, change the “soil” on which tumors depend, and can be organically combined with antitumor cell therapy to improve the treatment of malignant tumors (Chen et al., 2019). For example, polymer nanoparticles, liposome nanostructures, carbon-based nanomaterials, and metal nanoparticles (such as gold and zinc oxide nanoparticles) can inhibit the function of tumor cells and exert antitumor effects by increasing oxidative stress-mediated apoptosis and autophagy (Abad Paskeh et al., 2022). Chemotherapy drugs often have insufficient effective contact with solid tumors or cumulative concentration in tumors, restricting their antitumor effects. Therefore, loading paclitaxel or platinum drugs on nanoparticles with stronger targeting can make up for the poor delivery efficiency and insufficient killing of small-molecule chemotherapy drugs. Moreover, small-sized nanoparticles show longer blood circulation time than large-sized nanoparticles, so the concentration in tumor tissue becomes relatively high. Therefore, nano-modification of antitumor drugs is a necessary means to improve targeting and permeability and has broad research prospects.

#### Drug delivery for infectious diseases

Viral infections are still a worldwide public health problem. Liposome nanoparticles, micelles, dendrimers, and nanocapsules can be used to enter the human body through oral, direct injection, and inhalation routes. They can promote the potential of viral preparation for treatment and protection, and can be used for the treatment of AIDS (Oti, 2020), hepatitis B virus (HBV), and tuberculosis. Tuberculosis (TB), one of the deadliest diseases in the world, is caused by *Mycobacterium tuberculosis* (MTB). MTB invades host macrophages and other immune cells, modifies its lysosomal transport protein, prevents the formation of phagocytic lysosomes, and inhibits TNF receptor-dependent apoptosis in macrophages and monocytes. With the emergence of drug-resistant strains, it is increasingly difficult to cure tuberculosis. Polymer nanoparticles carrying antituberculous drugs can eradicate and control drug-sensitive and drug-resistant *M. tuberculosis* strains (Bashir et al., 2021), effectively improving the cure rate of tuberculosis. Near-infrared-responsive biomimetic nanoparticles (UCNPS)-Cas9@CM can effectively deliver Cas9 RNP and realize effective HBV treatment genome editing, which shows that the bionic nano-platform based on upconversion nanoparticles (UCNPs) can inhibit HBV replication through CRISPR treatment, and is a potential system for effective treatment of human HBV infection (Wang Dan et al., 2022). Gather (β-amino ester) polymer can be complexed with mRNA to form nanoparticles that can be used to prevent SARS-CoV-2 infection (Cristina et al., 2021). In addition, encapsulation of virus mRNA into appropriately sized cationic lipid nanoparticles (LNPs) prevents the imaging of extracellular ribonuclease and promotes the uptake and release of mRNA from the target cell endosomes (membrane-bound vesicles that are involved in the transportation and sorting of internalized material) (Reichmuth et al., 2016). A novel LNP delivery system was used to treat SARS-CoV-2 infection. Multiple siRNAs targeting the highly conserved region of the SARS-CoV-2 virus were screened. Three candidate siRNAs appeared. They were encapsulated in these LNPs and injected *in vivo*. With monotherapy or combination therapy, they both inhibited more than 90% of the SARS-CoV-2 virus effectively (Idris et al., 2021). Nanoparticle-based vaccines that deliver SARS-CoV-2 antigens will play an increasing role in extending or improving vaccination outcomes against COVID-19. More than 26 nanoparticle vaccine candidates have advanced into clinical testing (Vu et al., 2021).

#### Drug delivery for autoimmune diseases

Autoimmune disease is an immune regulation disorder. The self-tolerance mechanism is destroyed, resulting in the injury of self tissues and organs or the immunopathological state of abnormal function. The recovery period is long, and drugs or biological agents with extensive inhibitory effects are usually used for treatment. Therefore, the targeting is poor, the therapeutic effect is not obvious, and it is easy to produce side effects. The use of synthetic nanoparticles, biomimetic nanoparticles, and extracellular vesicles can improve drug targeting, have immunomodulatory properties, and can be used in the treatment of autoimmune diseases (Wen et al., 2021). Nanoparticles targeting T cells can be formulated into tolerant artificial antigen-presenting cells to correct the deficiency of homeostasis regulation and regenerate the production of therapeutic antigen-specific Treg, restore and maintain the advantage of Treg on effector cells, promote the long-term remission of autoimmune diseases, and finally prevent the emergence of the condition in susceptible individuals (Horwitz et al., 2021). Such substances can be used to treat systemic lupus erythematosus (SLE) (Zhang et al., 2020) and other autoimmune diseases. Psoriasis is a complex autoimmune disease. Mung bean-derived nanoparticles (MBNs) can improve the skin inflammation of psoriasis and regulate the immune microenvironment by regulating the skin immune system. For psoriatic skin stimulated by imiquimod (IMQ), local administration of MBNs can stabilize the internal environment of polarized macrophages and antagonize the NF-κB signaling pathway, reducing skin inflammation (Han et al., 2021).

#### Drug delivery for other diseases

Studies have shown that the complex drug delivery barrier in the eyes reduces the bioavailability of many drugs, resulting in poor therapeutic effects. When using nanocarriers to develop ocular drug delivery technology, the interaction between nanoparticles and ocular mucosa can prolong the retention time of drugs in the eyes and increase permeability (Zhang J. et al., 2021). The skin is the largest organ of the human body and the first line of defense against environmental insult. In a malnourished environment, skin must recover limited resources through the autophagy mechanism to maintain homeostasis. Polymer nanoparticles can deliver autophagy-related molecules (such as inducers, inhibitors, or nucleic acid molecules) to the skin and obtain better clinical therapeutic effects in immune-related skin diseases (Liu C. et al., 2021). Glycyrrhizic acid is a compound extracted from the common herb *Glycyrrhiza glabra*, which has mucosal protection, antioxidant, and anti-inflammatory effects. Glycyrrhizic acid-loaded polylactic acid glycolic acid (GA-PLGA) nanocarrier has a significant therapeutic effect on inflammatory bowel disease and can enhance the mucosal protection, anti-inflammatory, and antioxidant effects of 5-FU-induced intestinal mucositis (Zeeshan et al., 2021).

**FIGURE 3:**
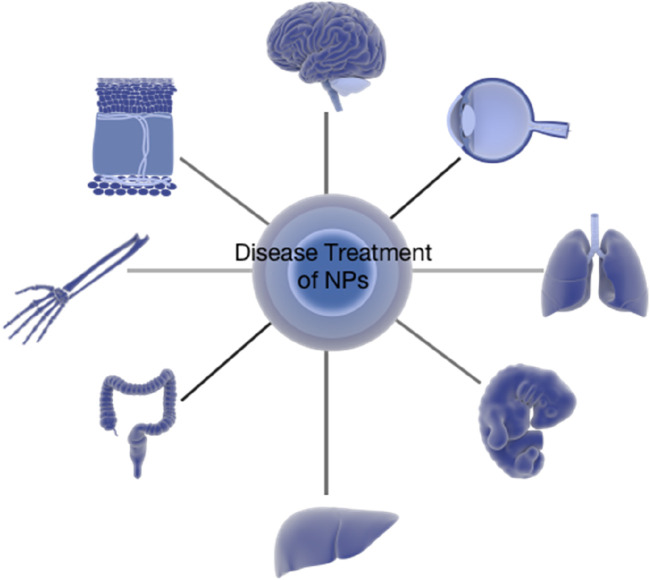
Disease treatment of nanoparticles. They can be used for diseases with a complex barrier effect (skin, placenta, or blood–brain barrier) because of their high targeting and penetration.

In summation, nanoparticle-based drug delivery systems can load substances such as small molecular drugs, plant extracts, peptides, or nucleic acids for therapeutic purposes. At the same time, nanoparticles can be effectively modified to change the lipid solubility and targeting of carriers for use in the treatment of a variety of diseases. With the continuous development of the biopharmaceutical field, biological macromolecules represented by antibodies, CAR-T, and antibody drug conjugate (ADC) have emerged. Because of their high targeting, strong cytotoxicity, fewer side effects, and high safety, compared with the difficulty of screening candidate targets of chemical drugs in early research and development, antibody-related drugs only need to prepare and verify antibodies against specific antigen epitopes, which are widely used in the treatment of tumors, autoimmune diseases, and other diseases (Figure 3).

## Characteristics and application of monoclonal antibodies

### Molecular characteristics and preparation of a monoclonal antibody

A monoclonal antibody (mAb) is a typical macromolecular drug. It refers to the highly uniform antibody produced by the cloning of a single B cell (its gene can only encode one antibody) and only targeting a specific antigen epitope. As a macromolecular protein, due to its special structure and physiological properties, its absorption, distribution, metabolism, and excretion *in vivo* are quite different from those of small-molecule drugs. It has unique pharmacokinetic characteristics such as target-mediated drug disposal, nonlinear pharmacokinetic metabolism, time dependence, and long half-life. mAb drugs are limited by their instability in the gastrointestinal tract (the influence of pH and digestive enzymes), poor fat solubility, large molecular weight, and limited intestinal permeability. They are usually administered by parenteral routes, including intravenous injection, subcutaneous injection, or intramuscular injection. Intravenous injection is the most common way of administration. The drug directly enters the blood without undergoing an absorption process and can act quickly (Ranchi et al., 2019). Intramuscular or subcutaneous injection of mAb drugs causes them to be absorbed through the human lymphatic system. The absorption rate is relatively slow. It generally takes 1–8 days to reach the maximum plasma concentration, and the bioavailability is generally approximately 50%–100% (Ovacik and Lin, 2018). Due to the large molecular weight and strong hydrophilicity, it is difficult for the drugs to diffuse freely into the tissue. The distribution in the tissue is very slow, and the distribution volume is usually very small. Therefore, mAb drugs are generally limited to the space of blood vessels and interstitial tissue. After intravenous injection, the distribution of mAb drugs from the vascular space to the interstitial space mainly occurs through convection (fluid flow from blood to interstitial space) and binding between antibody cells or receptor-mediated endocytosis, phagocytosis, and liquid pinocytosis (Glassman and Balthasar, 2019). mAb drugs are primarily eliminated by proteolytic enzyme catabolism, which produces smaller peptides and amino acids that are metabolized and eliminated in the form of energy substances or protein synthesis raw materials (Kamath, 2016). The elimination pathway can be divided into specific clearance and nonspecific clearance, which includes targeted-mediated clearance, nonspecific pinocytosis, and Fc receptor-mediated clearance (Deng et al., 2012).

The complete antibody molecule has three functional components: two fragment antigen-binding domains (FABs) and a fragment crystallization domain (Fc). The two FABs are connected to the Fc through a hinge region, which gives the FAB a great degree of conformational flexibility relative to the Fc. The FAB segment is an antigen-binding site, which contains a complementarity-determining region (CDR) that can complementarily bind to the corresponding antigen epitope. The Fc area can be connected with the Fcγ receptor (Fcγ r). It binds to the first subcomponent (C1q) of the C1 complex, mediates antibody-dependent cell-mediated cytotoxicity (ADCC), complement-dependent cytotoxicity (CDC), antibody-dependent cellular phagocytosis (ADCP), ectopic phagocytosis (trogocytosis), and induction of mediator secretion, and regulates tissue and serum half-life through interaction with FcRn. The Fc has long been the focus of important engineering to regulate the activity of the effector function, which is found in monocytes, macrophages, dendritic cells, neutrophils, T and B lymphocytes, and natural killer (NK) cells. The hinge area is located between the CH1 and CH2 functional areas. This region is rich in proline, so it is easy to stretch and bend. It can change the distance between the “Y” arms, which is conducive to the simultaneous combination of two identical antigen epitopes. It is also conducive to the exposure of complement-binding sites of immunoglobulin molecules and activates the complement by binding with complement C1q.

At present, many monoclonal antibodies can be prepared by hybridoma technology or a phage antibody library through the following mechanisms: antibody-dependent cell-mediated cytotoxicity (ADCC), complement-dependent cytotoxicity (CDC), inhibiting the binding of virus and cell receptor, inhibiting cell proliferation, regulating cell interaction, neutralizing antigens, and other mechanisms to prevent pathogenic substances from invading cells and treat infections and other related diseases.

### Clinical application of monoclonal antibody

Monoclonal antibodies can be divided into five subtypes according to the difference in molecular size and function: IgG, IgM, IgE, IgA, and IgD. Generally, most therapeutic antibodies are IgG. At present, mAb has been widely used in ELISA, radioimmunoassay, immunohistochemistry, flow cytometry, protein purification, and targeted therapy, such as the treatment and diagnosis of infectious diseases, autoimmune diseases, tumors, and other fields (Scheffel et al., 2022).

#### Treatment of infectious diseases with monoclonal antibodies

Monoclonal antibodies can be used to treat infectious diseases such as malaria, HBV, and COVID-19. Malaria is an insect-borne disease caused by a female *Anopheles* mosquito biting or injecting blood carrying *Plasmodium* in the infection stage. Particularly in some countries in Africa, Southeast Asia, and Central and South America, the mortality rate of falciparum malaria is very high. CIS43LS is an antimalarial monoclonal antibody with an extended half-life. The use of long-acting monoclonal antibody CIS43LS after infection control can prevent malaria (Gaudinski et al., 2021). Hepatitis B is caused by the hepatitis B virus (HBV). Hepatitis B patients and HBV carriers are the main sources of infection. HBV can be transmitted through mother and baby, blood, blood products, damaged skin and mucosa, and sexual contact. After HBV infection, liver fibrosis, cirrhosis, and even liver cancer may occur. In addition to hepatitis B vaccination, hepatitis B can also be treated by injecting monoclonal antibodies targeting HBV or transporting polypeptide factors. The phase IIa clinical trial conducted by Ascletis Pharma confirmed that ASC22 (envolizumab) showed good safety and tolerability for hepatitis B treatment. It is a PD-L1 monoclonal antibody. Blocking the PD-1/PD-L1 pathway can restore HBV-specific T-cell function and realize the functional cure of chronic hepatitis B. HBV also binds to the Na/taurocholate cotransporter polypeptide (NTCP) and enters host cells through the pre-S1 domain of the virus L protein. Therefore, NTCP should become the key goal of the development of anti-HBV therapy. A monoclonal antibody n6hb426-20 targeting NTCP, which can recognize the extracellular domain of human NTCP, prevents HBV from entering human hepatocytes and prevents HBV infection, but has little inhibitory effect on bile acid uptake. The advantage of NTCP targeting HBV entry inhibitors is that they remain effective regardless of virus genotype, virus mutation, and the presence of subviral particles (Takemori et al., 2022).

COVID-19 is caused by the severe acute respiratory syndrome coronavirus 2 (SARS-CoV-2). WHO data show that, to date, nearly 500 million people have been diagnosed worldwide, more than 6 million people have died, and more than 10 billion people have been vaccinated against the coronavirus (WHO, 2022). The viral spike (S) protein mediates viral attachment, entry into host cells, and serves as a critical target for vaccine design (Shang et al., 2020). The S protein is a kind of trimeric transmembrane glycoprotein composed of the S1/S2 heterodimer. It can recognize host cell receptor angiotensin-converting enzyme 2 (ACE2) and mediate membrane fusion. A C-terminal receptor binding domain (RBD) contained in the S protein is directly involved in the recognition of the host receptor and mediates virus invasion into host cells. Delivery of the S protein or the S-encoding nucleic acid as a vaccine induces neutralizing antibodies and antiviral T- and B-cell memory (Tian et al., 2021). The SARS-CoV-2 virus is prone to mutation, and different SARS-CoV viruses have different affinities to human ACE2, as well as different infectivity and transmissibility. This creates a huge challenge for the research and development of SARS-CoV-2 vaccines (Wang R. et al., 2021). The current COVID-19 therapeutic drugs include micromolecule antiviral drugs (remdesivir and ritonavir), anti-inflammatory drugs (tocilizumab and siltuximab), neutralizing antibodies with high specificity and low side effects (cocktail therapy, regn-cov2, regeneron/Ly-cov555, Lilly × AbCellera/VIR-7831, and VIR × GSK), and various vaccines: recombinant protein vaccine, nucleic acid vaccine, virus vector vaccine, inactivated vaccine and live attenuated vaccine. Induction of neutralizing antibodies (nAbs) was recently demonstrated to be a robust correlate of protective efficacy for current COVID-19 vaccines (Khoury et al., 2021). At present, there are three kinds of products officially used in China: inactivation, adenovirus vectors, and recombinant proteins. After vaccine administration, viruses produce multiple antibodies by recognizing epitopes. Most of them send signals to T-lymphocytes to lock in antigens, stimulate cellular immune response, and kill viruses. The neutralizing antibody is an antibody produced only against the virus-neutralizing epitope, which can directly target the virus-neutralizing epitope and make the virus lose the binding ability of its receptor, rather than activate the T-lymphoid system. Neutralizing antibodies also have the potential to prevent the virus from infecting the target cells. There are some advantages of monoclonal antibodies: a clear mechanism, and they are easy to produce on a large scale, which will become the key direction to preventing COVID-19. Studies have shown that the humanized COVID-19 monoclonal antibody can neutralize the SARS-CoV-2 mutant strain (Tan, 2022). A neutralizing monoclonal antibody targeting COVID-19 can be inhaled through a vibrating screen atomizer. As an early intervention measure after a positive diagnosis, it can significantly inhibit the development of COVID-19 (Lai et al., 2021). In a multicenter, double-blind, phase III clinical trial, solanezumab showed a low incidence of adverse reactions and significantly reduced the disease progression of COVID-19 (Gupta et al., 2021). In November 2021, the EU approved Regkirona (regdanvimab, CT-P59) for the treatment of adult COVID-19 patients who do not need an oxygen supplement and have an increased risk of developing serious diseases. At the same time, the Roche/Regeneron meta-antibody cocktail therapy, Ronapreve (casirivimab and imdevimab), has also been approved by the EU for the treatment of COVID-19. Monoclonal antibodies have gradually become the most promising treatment for targeting COVID-19 (Jahanshahlu and Rezaei, 2020).

#### Treatment of tumors with monoclonal antibodies

Monoclonal antibodies (mAb) have long been an important means of treating hematological malignancies or solid tumors. Clinically, many monoclonal antibodies are used in the treatment of hematomas and solid tumors. To date, more than 100 antibody drugs have been approved by the FDA. Among them, antitumor monoclonal antibodies include Pembrolizumab targeting PD-1 and navulizumab, which are used in the treatment of advanced gastric cancer and esophageal cancer (Joshi et al., 2018), cervical cancer (Marret et al., 2019), colorectal cancer (Smith and Desai, 2018), hepatocellular carcinoma (Finkelmeier et al., 2018), metastatic melanoma (Koppolu and Rekha Vasigala, 2018), and other solid tumors. Rituximab, targeting CD20, is often used in the treatment of non-Hodgkin lymphoma, diffuse large B-cell non-Hodgkin lymphoma, follicular lymphoma, and leukemia (Salles et al., 2017). HER-2 targeted pertuzumab and trastuzumab are used in the treatment of HER-2-positive breast cancer. Bevacizumab targeting VEGF is used in the treatment of various metastatic cancers. Many more monoclonal antibodies targeting tumors are in clinical trials. Researchers have developed an anti-ADAM17 monoclonal antibody that can play a significant therapeutic role in many solid tumors, including breast cancer, ovarian cancer, glioma, colon cancer, and lung adenocarcinoma (Hassani et al., 2021; Saha et al., 2022). Even the anti-CD33 antibody drug conjugate has improved the survival rate of patients with acute myeloid leukemia (Linde and Walter, 2019), and anti-CD47 and anti-CD70 antibodies have similar effects (Venugopal et al., 2021).

The development of antitumor monoclonal antibody drugs is still the main track for the research and application of monoclonal antibodies in the future, although most focus on immune checkpoints such as PD-1/PD-L1, CTLA-4, or other targets. With the development of high-throughput sequencing, there will be more specific new targets to be discovered and studied deeply in the future.

#### Treatment of autoimmune diseases with monoclonal antibodies

A variety of monoclonal antibodies have been listed for the treatment of autoimmune diseases. For example, belimumab, developed by GSK, is the first biological agent approved for the treatment of systemic lupus erythematosus (SLE). It is a specific human IgG1 mAb of soluble B-lymphocyte stimulator (BLyS). Intravenous administration can inhibit the survival of B cells and cause more autoreactive B cells to undergo apoptosis by blocking the binding of soluble BLyS to the receptor on B cells, thereby reducing the autoantibodies in serum. At the same time, it does not affect the cells in the late stage (such as memory B cells or plasma cells that survive for a long time), and retains the immunity. In 2015, the US FDA approved Cosentyx (secukinumab) for the treatment of adult patients with moderate and severe plaque psoriasis.

#### Treatment of inflammatory diseases with monoclonal antibodies

Some monoclonal antibodies commonly used for anti-inflammatory therapy include adalimumab and infliximab targeting TNF-α, which are used to treat rheumatoid arthritis (Cacciapaglia et al., 2022) and psoriasis (Sbidian et al., 2017). Ocrelizumab, targeting CD20, can be used in the treatment of multiple sclerosis (Nielsen et al., 2021). Gastrocnemius myalgia syndrome (GMS) is a rare inflammatory myopathy associated with Crohn’s disease (CD). Infliximab targets anti-TNF-α treatment may be an effective treatment option (Catherine et al., 2022).

Although monoclonal antibodies have been successfully used in tumor treatment, their application in the development of treatment is still hindered due to the limitations of tumor penetration and high preparation costs. Clinically, monoclonal antibodies may have a low response rate and high drug resistance. Drug combinations are usually required. When a nano-drug delivery system is adopted, antibody fragments are nano-modified to improve their permeability and tissue distribution concentration, reduce side effects, and improve the therapeutic effect of the antibody.

### Nano-drug delivery systems for monoclonal antibodies

Since 1975, Kohler has successfully obtained mouse-derived monoclonal antibodies with antigen specificity through hybridoma technology, thus opening a new era of monoclonal antibodies. With the continuous development and application of genetic engineering technology and nanotechnology, the antibody drug delivery system is also constantly optimized and improved. It has gradually replaced the traditional antibody and become the mainstream of biopharmaceutical and medical diagnosis. Co-loading two or more drugs with different physical and chemical properties and pharmacological mechanisms into nanoparticles is called a nano-drug co-delivery system (NDCDS) (Wan et al., 2019), which has also attracted much attention this year. Therefore, nanobodies and micromolecule/mAb delivery nanoparticles have become a potential way to deliver a targeted drug.

#### Nanobody–drug co-delivery system

Studies have shown that anti-IgG (Fc-specific) antibody (αFc) coupled to the surface of nanoparticles (αFc NP) establishes a general antibody platform. The studies confirmed that αFc NP can easily and effectively immobilize different kinds of immune effector cells through Fc and form imNA through the non-covalent interaction of the specificity of mAb. It is verified that imNAs play a superior role than the parent mAb mixture in antitumor immune response that is mediated by T cells, NK cells, and macrophages in a variety of mouse tumor models (Jiang et al., 2021). An anti-TLR4 nanoantibody obtained by phage display technology can effectively reduce the release of inflammatory factors and improve the survival rate of animals. When the C-terminal and intermediate domains are closed at the same time, the effect is more obvious and can be used for the treatment of sepsis (Liao et al., 2021). According to the latest research, broad and potent neutralizing llama single-domain antibodies (VHH) against HIV-1 targeting the CD4 binding site (CD4bs) that have previously been isolated upon llama immunization (Strokappe et al., 2019) have a better treatment effect on HIV. The mAb nano-drug delivery system (NDDS) mAb improves the affinity of drugs and can capture the virus before it binds to human ACE2. Therefore, it can be used as a drug carrier to prevent and treat COVID-19 (Abduljauwa et al., 2020).

In the clinic, a nano artificial antibody is used to specifically recognize circulating tumor cells (CTCs). Under the same conditions, the ability of artificial antibodies to recognize CTCs is eight times that of white blood cells (Zhang et al., 2017). Nanotechnology has made rapid progress in assisting vaccine development. The method can be used to deliver nucleic acid and conformationally stable subunit vaccines to regional lymph nodes and trigger effective humoral and cellular immunity to prevent viral infection or control the severity of the disease (Nel and Miller, 2021). Nanoparticles can be loaded with multiple targets (e.g., antigens, epitopes, and adjuvants) and surface modified (e.g., targeted ligands or surface coatings) to promote the entry into lymph nodes and germinal centers (Pardi et al., 2018). A novel albumin nanoparticle named EGa1–PEG–anti-EGFR nanobody showed a 40-fold higher binding to EGFR-positive 14C squamous head and neck cancer cells (Altintas et al., 2013).

In order to achieve sufficient therapeutic effect, the dose of antibody drugs must be higher than that of conventional drugs, but this is difficult to achieve in practice. Using nanocarriers to bind antibodies or fragments to reduce the dose, targeted immobilization can enhance their affinity, endocytosis efficiency, and therapeutic effect (Iijima et al., 2019). In addition, the NDDS can also be used to achieve excellent performance, such as targeted drug delivery, tumor microenvironment response, and controlled drug release (Mu et al., 2020). It has broad application prospects in the field of tumor immunotherapy. Some studies have shown that the surface of a cross-linked starch iron oxide nanocarrier combined with a specifically modified CD11c monoclonal antibody has a good affinity for mouse dendritic cells. At the same time, it can show the directional effect of monoclonal antibody and good targeting (Brückner et al., 2021). Chitosan-based nanocarriers (CS-NCs) establish ion interaction with endothelial cells, promote drug transport through the BBB through adsorption-mediated cell transport, and are used in the treatment of brain tonic diseases, especially tumors (Caprifico et al., 2020). Targeting cell adhesion molecule (CAM)-mediated endocytosis induced by intercellular adhesion molecule-1 (ICAM-1) and combining anti-ICAM-1 antibody with polymer nanocarriers (NCs) is also conducive to penetrating the BBB and treating brain tumors (Anselmo et al., 2015). Superparamagnetic iron oxide nanoparticles were synthesized with anti-IL-4R through polyethylene glycol polymer α blocking antibody (SPION-IL4Rα) can inhibit the development of breast cancer and can also be combined with antitumor antibiotics to improve the therapeutic effect (Pasha Shaik et al., 2017). By coupling the anti-HER-2 to the adriamycin (DOX) magnetic dextran spermine (DEX-SP) nanoparticle carrier (DEX-SP-DOX), the cell internalization is increased, which is beneficial to the treatment of HER-2-positive breast cancer (Tarvirdipour et al., 2016). The semichain fragment of anti-EGFR monoclonal antibody (cetuximab) is combined with colloidal nanoparticles to produce stable nanoconjugates, which can be used as a substitute for therapeutic monoclonal antibody for triple-negative breast cancer (TNBC). The nanoscale conjugation of this half-chain monoclonal antibody can improve the therapeutic effect, enhance the antibody activity, and improve the targeting selectivity of TNBC cells and can also be used for the treatment of drug-resistant tumors (Colombo et al., 2018). Macrophage targeting and phagocytosis-induced DDS nanocarriers can deliver monoclonal antibodies to the surface of macrophages, which can be effectively absorbed by macrophages by endocytosis (Li et al., 2018). This also provides a new insight for the nano-delivery of monoclonal antibodies targeting the tumor microenvironment (TME).

#### Nanobody-based drug delivery system

A nanobody (NB) is a single-domain antibody. This kind of antibody contains only one heavy-chain variable region (VHH) and CH2 and CH3 regions. Traditional antibodies have two variable domains, called VH and VL, which provide stability and binding specificity to each other. Compared with other antibodies, the light chain is missing (Zhang et al., 2018). The VHH crystal is 2.5 nm wide and 4 nm long. It is the smallest fragment that can bind to antigens naturally. There are only approximately 10 amino acids on its surface, which is different from human VH, and it has four specific amino acids: FR2: V37, G44, L45, and W47. In the common FR2 antibody, there are hydrophobic residues that are quite conserved in evolution, while in VHH, these four amino acid residues are mutated into hydrophilic amino acid residues F37, E44, R45, and G47, which increase the water solubility of VHH and eliminate solubility and aggregation problems. With this characteristic, the VH sequence of the human antibody can be optimized, and some amino acids in FR2 can be modified to improve the stability and solubility of the VH antibody and maintain the original specificity and affinity. Because the nanoantibody lacks an Fc segment, it avoids the complement reaction caused by Fc, resulting in good biocompatibility.

**FIGURE 4:**
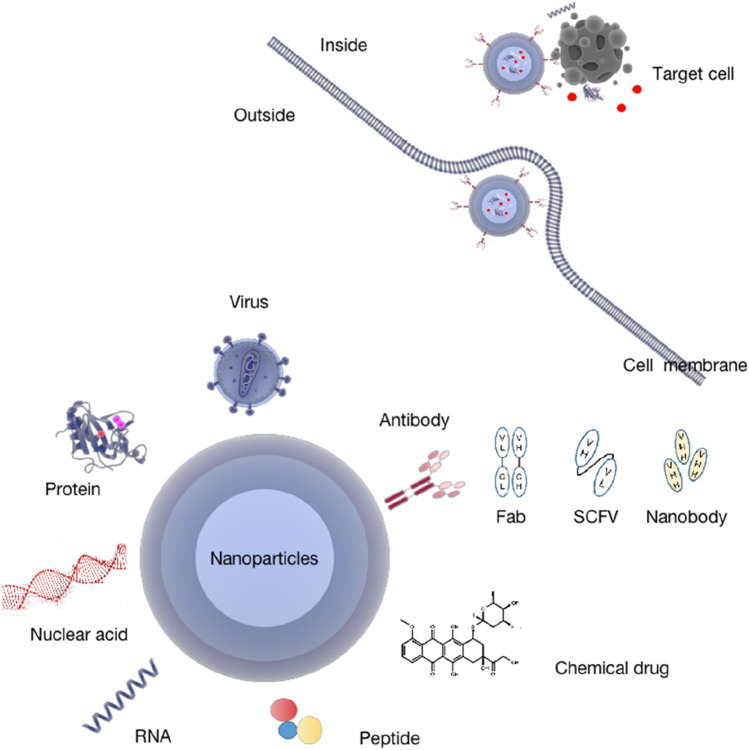
Nano-drug delivery systems for antibodies. Nanoantibodies penetrate the barrier and reach the target organs, enrich the target tissue, mediate apoptosis and autophagy through several effects, and regulate the immune microenvironment to achieve their therapeutic effect.

VHH also has strong antigen targeting and binding ability and offers many advantages compared to ordinary antibodies (Surendra Panikar et al., 2021). For example, it has a low molecular weight and can enter parts of the body that cannot be reached by full-length antibodies. Only 15-kDa nanoantibodies can penetrate the blood–brain barrier, providing a new method for the research and treatment of diseases in the brain. It can adapt to different routes of administration and is more beneficial to industrial production. Unlike traditional antibody fragments, despite 80 °C temperature and extreme pH, it can still exist and work stably with completely reversible deployment. Compared with full-length antibodies, it is only 1/10 of the molecular weight of the nanoantibody, along with reduced humoral and cellular immune response and specific antibody, causing lower immunogenicity. However, nanobodies have a short half-life period. Genetic engineering methods can be used to address this question, such as Fc fusion, PEG, or albumin fusion to satisfy the demand of drug delivery (Bever et al., 2016). Because of the single domain, it can be easily coupled with other molecules, such as connecting radioisotopes, toxins to prepare immunotoxins, or form new fusion molecules with other structures through genetic engineering (such as enzymes and antimicrobial peptides) to prolong the half-life. Nanobodies are also suitable for targeting intracellular and even nuclear proteins as intracellular antibodies. Studies have shown that the HEMA nanobody has significant penetration into living cells (Wei et al., 2021). A nanoantibody targeting a tumor can penetrate the barrier, reach the target site, and remain for a sufficient time to obtain a better synergistic antitumor effect (Peng et al., 2020). Using nanoparticles in a drug delivery system can promote the circulation cycle, reduce toxicity (Wang Yingli et al., 2022), and promote the therapeutic effect of the antibody. Therefore, a nanobody-mediated drug-targeted delivery system is more suitable for drug delivery routes than conventional monoclonal antibodies (Liu Manman et al., 2021) (Figure 4).

Caplacizumab, the world’s first nanobody, is a humanized nanoantibody with strong selectivity for bivalently anti-vascular Willebrand factor (vWF). It can block the interaction between the very large vWF polymer and platelets, has an immediate effect on platelet aggregation, and also affects the subsequent formation and accumulation of micro blood clots. It was approved in 2018 for the treatment of acquired thrombotic thrombocytopenic purpura (aTTP). Because there is no treatment drug for the disease on the market, it became the first drug in its class and qualified as an orphan drug. Several other nanobodies are currently in clinical research. These are distributed in tumors (Katerina et al., 2021), neurological diseases (Zhang W. et al., 2021), infectious diseases (Hanke et al., 2022), skin diseases (Rubel, 2021), and immune system diseases (Papp et al., 2021).

### Perspective

On the one hand, due to the special pharmacokinetic/pharmacodynamic (PK/PD) properties of biological macromolecules, the limited route of administration leads to a low drug absorption rate, difficulty in distribution in target organs or tissues, difficulty in reaching the blood drug concentration, and poor drug permeability (Thiramanas et al., 2020). On the other hand, micromolecule drugs have a long research cycle because of numerous candidate compounds and less target specificity. Nanoparticles combine the advantages of these two: wide application in drug delivery systems (Mukherjee et al., 2019), especially in antibody delivery, improved safety, effectiveness, targeting, and permeability (Li et al., 2019), crossing the human body barriers (blood–brain barrier, skin–mucosal barrier, and placental barrier), reach, and continuously enrich in the target position. They mitigate the limitations of conventional antibodies, such as high immunogenicity, low stability, few delivery routes, high apparent cost, and unfavorable industrial production. Nanoparticles can be loaded with different toxins and targeted components and then modified with liposomes, polymer micelles, and nanometals to change the pharmacokinetic characteristics of drugs, regulate the immune microenvironment, and control drug release (Terstappen et al., 2021). This shortens the research cycle, making them very suitable for the requirement of targeted drug delivery in modern medicine (Table 1).

**TABLE 1:** Comparison of nanocarrier types for antibody/nanobody delivery.

Nanocarrier type	Advantage	Limitation
Liposomes	High biocompatibility, ability to encapsulate hydrophilic/hydrophobic drugs, and FDA-approved formulations available	Rapid clearance, stability issues, and potential immunogenicity
Polymeric nanoparticles	Controlled release, tunable degradation, and surface functionalization	Possible polymer toxicity and batch-to-batch variability
Metal nanoparticles	Unique optical/magnetic properties, photothermal therapy, and imaging contrast	Potential metal toxicity, long-term accumulation, and cost
Lipid nanoparticles	High mRNA/DNA encapsulation efficiency, endosomal escape, and scalable production	Liver tropism, reactogenicity, and storage stability
Protein/albumin nanoparticles	Natural targeting biodegradability and low immunogenicity	Limited drug loading and stability in circulation
Dendrimers	Monodisperse, multivalent surface, and high drug loading	Synthesis complexity and potential cytotoxicity

Nano-drug delivery systems (NDDS) are progressively demonstrating their potential as valid alternatives or complements to conventional monoclonal antibodies for clinical applications. They serve not only as carriers for monoclonal antibodies but also for other macromolecular therapeutics, including antibody–drug conjugates (ADCs) and chimeric antigen receptor T-cell (CAR-T) therapies. Notably, nanobodies and smaller antigen-binding fragments are significantly smaller than full-length IgG, enabling deeper penetration into solid tumors. When integrated into CAR-T therapy or into ADCs, nanobodies can improve specificity through multi-epitope targeting, potentially reducing side effects. This approach represents a promising mainstay strategy for treating diseases with complex microenvironments.

However, translating nanoparticle-based antibody delivery systems faces several significant challenges that must be urgently addressed. While nanobodies offer advantages, concerns regarding their immunogenicity and potential renal clearance remain. More broadly, the safety profile of engineered nanocarriers demands rigorous evaluation. Key issues include

Biodegradability and long-term fate: Some polymeric or inorganic nanoparticles may exhibit slow or incomplete degradation *in vivo*, leading to potential long-term accumulation in organs such as the liver, spleen, and lymph nodes, raising concerns about chronic toxicity (Parodi et al., 2021). Immunotoxicity and immune activation: Beyond the desired therapeutic effects, nanoparticles can unintentionally activate the immune system, leading to a complement activation-related pseudoallergy (CARPA), inflammation, or unforeseen immunomodulatory consequences that could undermine therapeutic efficacy or safety (Mitchell et al., 2021). Batch-to-batch variability and scalability: Reproducible synthesis of nanoparticles with uniform size, surface charge, and drug loading remains a manufacturing hurdle, especially for complex multi-component systems like lipid nanoparticles (LNPs). This variability can directly impact pharmacokinetics and therapeutic outcomes (Vargas et al., 2025). Targeted delivery efficiency and off-target effects: Although enhanced permeability and retention (EPR) effect and active targeting strategies improve tumor accumulation, a significant portion of administered nanoparticles still ends up in non-target organs, particularly the mononuclear phagocyte system (MPS), which could lead to off-target toxicity (Rosenblum et al., 2025).

Future research directions should, therefore, focus on the rational design of “smarter” nanoparticles with improved biocompatibility, controlled degradation profiles, and enhanced target specificity. Developing robust characterization standards and predictive toxicological models is crucial for clinical translation. Exploring combination strategies where nanotechnology synergizes with other treatment modalities will likely yield the next-generation transformative therapies.

To sum up, the development and application of the nanobody delivery system will drive future advances. Given the enormous research activity in the field, it can be expected that increasing numbers of nanobodies and their conjugates will undergo late clinical application in the future.
